# Degradation of Drug Delivery Nanocarriers and Payload Release: A Review of Physical Methods for Tracing Nanocarrier Biological Fate

**DOI:** 10.3390/pharmaceutics13060770

**Published:** 2021-05-21

**Authors:** Patrick M. Perrigue, Richard A. Murray, Angelika Mielcarek, Agata Henschke, Sergio E. Moya

**Affiliations:** 1 NanoBioMedical Centre, Adam Mickiewicz University, Wszechnicy Piastowskiej 3, 61-614 Poznan, Poland; patrick.perrigue@amu.edu.pl (P.M.P.); angelika.mielcarek@amu.edu.pl (A.M.); agata.henschke@amu.edu.pl (A.H.); 2 Instituto Biofisika (UPV/EHU, CSIC), Barrio Sarriena S/N, 48940 Leioa, Spain; richard.murray@ehu.eus; 3 Center for Cooperative Research in Biomaterials (CIC biomaGUNE), Basque Research and Technology Alliance (BRTA), Paseo de Miramon 182, 20014 Donostia San Sebastián, Spain

**Keywords:** biological fate, degradation, drug delivery, molecular imaging, fluorescence microscopy, Raman microscopy

## Abstract

Nanoformulations offer multiple advantages over conventional drug delivery, enhancing solubility, biocompatibility, and bioavailability of drugs. Nanocarriers can be engineered with targeting ligands for reaching specific tissue or cells, thus reducing the side effects of payloads. Following systemic delivery, nanocarriers must deliver encapsulated drugs, usually through nanocarrier degradation. A premature degradation, or the loss of the nanocarrier coating, may prevent the drug’s delivery to the targeted tissue. Despite their importance, stability and degradation of nanocarriers in biological environments are largely not studied in the literature. Here we review techniques for tracing the fate of nanocarriers, focusing on nanocarrier degradation and drug release both intracellularly and in vivo. Intracellularly, we will discuss different fluorescence techniques: confocal laser scanning microscopy, fluorescence correlation spectroscopy, lifetime imaging, flow cytometry, etc. We also consider confocal Raman microscopy as a label-free technique to trace colocalization of nanocarriers and drugs. In vivo we will consider fluorescence and nuclear imaging for tracing nanocarriers. Positron emission tomography and single-photon emission computed tomography are used for a quantitative assessment of nanocarrier and payload biodistribution. Strategies for dual radiolabelling of the nanocarriers and the payload for tracing carrier degradation, as well as the efficacy of the payload delivery in vivo, are also discussed.

## 1. Introduction

Nanoparticles (NPs) offer multiple possibilities for the advancement of medicine, bringing solutions to problems that cannot be solved with standard medical approaches [1]. The use of nano- and microtechnologies in drug delivery provides many advantages for the encapsulation of otherwise insoluble therapeutics [2], for programmed release [3] and especially for cell and organ targeting [4]. Nanocarriers (NCs) have been designed for cancer treatment [5] and gene therapy [6], for the treatment of Alzheimer’s disease [7], and for the delivery of antibodies in rheumatoid diseases, to cite a few examples. Some of the developments in the nanomedicine field have reached the market, while others are presently in the clinical phase [8]. Transport and delivery of NCs and encapsulated drugs or cargo are strongly affected by the physicochemical characteristics of the NCs, such as their size, shape, surface charge, and chemical formulation. The most common materials employed in the manufacture of NCs are polymers [9], lipids [10,11], and metals [12]. Inorganic metal or metal oxide NCs offer several advantages due to their unique properties for imaging and therapy; however, they are limited by their toxicity, slow degradation, and rapid elimination when used for medical purposes [13]. An interesting type of inorganic NPs with potential for drug delivery are mesoporous silica NPs (MSNs), which can be loaded with cargo in nanoscale pores. Another example of porous materials with potential for drug delivery are metal–organic frameworks (MOFs), which can be considered hybrid materials formed by the complexation of organic linkers and ions to form three-dimensional networks [14].

Phospholipids formulated into liposomes are probably the most successful organic material used as a NC. It was in the early 1960s when liposomes were described for the first time, eventually becoming a powerful tool for drug delivery. Liposomes are vesicles formed by at least a single lipid bilayer. Multiple products based on liposomes have reached the pharmaceutical market, such as Doxil^®^ (breast cancer), Abelcet^®^ (fungal infections), or Marquibo^®^ (lymphoblastic leukaemia) [15]. Hydrophilic drugs can be encapsulated inside the aqueous phase cavity of the liposomes, while hydrophobic drugs can be embedded within the hydrophobic regions of the lipid bilayer. Lipid and also polymeric NCs can improve the therapeutic index of various water soluble/insoluble drugs by increasing their bioavailability, their solubility in physiological environments, and retention time in the tissue or organ of interest [16]. Biocompatible and biodegradable poly lactic-co-glycolic acid (PLGA) and poly lactic acid (PLL) NPs are examples of polymeric NCs for drug delivery that have reached clinics and which can be used for encapsulating hydrophobic payloads in their cores [17]. Another interesting example of NCs based on biopolymers are protein-based NCs, which are biodegradable, non-antigenic, metabolizable, and are easily amenable for surface modification [18]. Protein NPs are already in the market. These NCs are based on the assembly or covalent crosslinking of proteins, with bovine serum albumin (BSA) or human serum albumin (HSA) being the most common protein employed for this type of NC. The presence of hydrophobic and hydrophilic domains in the proteins make them suitable for encapsulation of multiple drugs as well as therapeutics. There are also polymer carriers based on the assembly of synthetic amphiphilic polymers in micelles with a hydrophobic core and a hydrophilic coating, spontaneously formed in aqueous media. In analogy to lipids, amphiphilic polymers can assemble into bilayers that close in on themselves, forming the so-called polymersomes [4,19]. Highly hydrated crosslinked polymer networks, called hydrogels, also in nano and micro, can also be used for drug or therapeutic encapsulation.

The determination of systemic and intracellular degradation and biological fate remains unexplored for most NCs, despite being fundamental for the successful transport and delivery of encapsulated drugs. The design of NCs often requires complex engineering of the NC surface, usually involving an external coating with a shell of polyethylene glycol (PEG), proteins, sugars, or zwitterionic molecules to prevent plasma protein binding and to enhance stability during circulation. The binding of plasma proteins is the primary mechanism for the mononuclear phagocyte system (MPS) to recognize the circulating NCs, causing a major loss of the administered dose; such effects can be dramatic following specific administration routes (e.g., intravenous injection) [20]. The “stealth” coating is also fundamental in preventing NC aggregation. The size of the NCs or the resulting aggregates will ultimately determine their fate in vivo. While large aggregates may create micro-emboli in the capillary vessels of the lungs and accumulate there, NCs with sizes in the range 100–500 nm tend to accumulate in the organs of the MPS, mainly the lungs, the liver, and the spleen. Hence, the stability of the coating during circulation is fundamental to prevent rapid clearance and to guarantee the accumulation of the drug in the targeted organs. Moreover, targeting functions, such as peptides, antibodies, or proteins, are attached to the surface of the NCs. Coating degradation would lead to the removal of these targeting functions and consequently their recognition and enhanced uptake in selected tissue and organs will be lost.

At the cellular level, polymer NCs are mainly visualized using Raman microscopy, confocal laser scanning microscopy (CLSM), and flow cytometry (FC). The latter two require the fluorescent labelling of the NCs. To address NC degradation and intracellular fate-related issues, the use and combination of sophisticated labelling strategies enabling discrimination between (i) the core and the coating, and (ii) different depths in the core, are required. NC degradation and aggregation can also be studied by fluorescence correlation spectroscopy (FCS), a technique based on recording fluorescence fluctuations in a confocal volume. FCS is based on the diffusion times of fluorescent molecules that correlate to the diffusing species’ size. Aggregation or degradation of labelled NCs lead to changes in the diffusion time of fluorescent species attached to the NCs [21].

Nanomaterial degradation, such as the loss of surface coatings or targeting functionalities, is often a burden for achieving NC targeting. However, it is also a means to deliver encapsulated drugs, which are often only delivered when the nanomaterial degrades or changes under specific environmental stimuli, such as a change in pH from the bloodstream to endosomes. If the cargo drug is retained inside the NCs, it may finally not be delivered. Colocalization studies of cargo and NCs bring fundamental information on the capacity of the NC to provide the drug in the desired manner. Moreover, the fate of the NCs and encapsulated drug must coincide until the desired tissue or cell for delivery. Tracing the fate of cargo and NCs in vitro can be achieved by fluorescently labelling the NCs and cargo with spectroscopically different dyes and tracking their spatial colocalization by CLSM. In some cases, if the cargo can be labelled with dyes sensitive to differences in polarity or other environmental conditions, it may not be necessary to label the NC, as from changes in the fluorescence of the dye it would be possible to discriminate between the encapsulated and free dye. Fluorescence lifetime imaging microscopy (FLIM) is particularly suitable for these studies as the lifetime of fluorescence dyes is highly sensitive to environmental conditions and usually changes if the dye is encapsulated in NCs or free in a cellular milieu.

Raman microscopy provides a means to track the fate of drugs and NCs in cells or tissue without any labelling [22,23], relying on intrinsic Raman spectra from both the NCs and drugs, which must not overlap completely with each other, and also with intrinsic Raman bands from the biological matrix where they are located for their simultaneous colocalization inside the cells.

Ex vivo and in vivo, NCs can be visualized using different imaging modalities involving fluorescence and radiolabelling. Nuclear imaging techniques, such as positron emission tomography (PET) and single-photon emission computer tomography (SPECT), have gained relevance as they provide a means to quantify NC per organ through activity measurements. Both techniques provide spatiotemporal information about the biodistribution of radiotags with unparalleled sensitivity and are non-invasive. Alternatively, fluorescent NCs can also be traced in small animals using sensitive cameras for in vivo detection. As for intracellular studies, tracing NC degradation in vivo requires sophisticated labelling strategies with dual or multiple labelling of the NC core and coatings. This review will focus on the physico-chemical and biophysical methods that can be used to trace NC degradation and release of encapsulated cargo in biological matrixes. Both aspects require ingenious strategies of labelling and the application of non-standard techniques for the characterization of NCs, especially in the case of in vivo studies. However, both NC stability and the interplay between NC and drug fate are fundamental for the assessment of the efficacy of carriers to deliver the cargo as planned, in targeted tissue and cells.

## 2. In Vitro Techniques

### 2.1. Confocal Laser Scanning Microscopy

CLSM is an optical imaging technique for obtaining high-resolution images with depth selectivity. CLSM uses an illumination system that directs laser light onto a focused spot in a sample, scanning point-by-point over an area and recording each spot’s intensity. The laser light hits fluorescing molecules, and these molecules emit light at lower energies that are picked up by a detector, usually a CCD camera. The optics inside the microscope eliminate parts of the sample that are not in focus, accomplished by a pin-hole, which only allows light from the in-focus region. The result is an image of a thin focal plane, otherwise known as an optical section. CLSM overcomes the fundamental problem of blurriness associated with conventional fluorescence microscopy. The scanning of multiple areas at different depths combined with computer calculation allows for 3D reconstruction of a biological sample.

Tracing NCs in the cell is essential for evaluating their performance for drug delivery. CLSM can visualize NCs as they translocate with time, close to the cell membrane, endosomes (early and late), and in the endoplasmic reticulum [24,25,26]. NCs must be fluorescently labelled for their tracing by CLSM. Labelling can take place on the surface of the carriers through conjugation of a fluorescent dye to the NC, or by entrapment of the dye inside the NC. The first option is probably the most common approach. However, if the dye detaches from the NC, this will result in a false indication of the NC localization [27]. The labelling of cell compartments, namely, the nucleus, mitochondria, endosomes, cell membranes, etc., with spectrally distinct dyes allows for colocalization measurements. CLSM is a useful tool to visualize trafficking of the NCs and drugs [28].

As previously stated, the degradation of NCs depends on their interaction with biomolecules, changes in pH and ionic strength, and the action of enzymes. CLSM can be used to trace NC degradation by labelling different components of the carriers with spectrally distinct dyes, i.e., labelling the core and coating. The degree of overlap of the two dyes would provide information on the stability of the NC. If the NC degrades, or the dye on the coating detaches, colocalization will decrease. The detachment of the dye per se is not necessarily indicative of degradation as it may not mean that the molecules to which the dye is attached will also be released. Therefore, dual labelling studies must be carefully planned or CLSM applied in combination with other techniques, such as the Förster resonance energy transfer (FRET), FCS, or FLIM. 

Di Silvio et al. studied the intracellular trafficking of NPs prepared by complexation of poly allyl amine hydrochloride (PAH) and silencing RNA (siRNA), which were designed for gene therapy [29]. For gene silencing to take occur, the complexes have to break, and the siRNA must be liberated in the cytoplasm. After cellular uptake, complexes are translocated in endosomal compartments with more acidic pH values, which favors their spontaneous dissociation and the diffusion of siRNA in the cytoplasm. Complexes were prepared with PAH labelled with green rhodamine and siRNAs labelled with the Cy5 dye. Green rhodamine and Cy5 show no overlapping in their fluorescence emission and the stability of PAH and siRNA as complexes can be evaluated from the colocalization of the fluorescence of the two dyes. CLSM of cells exposed to the NCs was performed after 1 h and 24 h of incubation in controlled conditions. Colocalization experiments performed at different time points in the cells confirmed a significant decrease in the co-localization between the red and green channels, corresponding to Cy5 and green rhodamine, respectively. After 24 h, Pearson’s coefficient, r, and Mander’s coefficients, M1 and M2, decreased by about 50%, suggesting that the complexes were dissociated. Pearson’s coefficient indicates the degree of linear correlation between the red and green channels, while Mander’s coefficients give the percentage of co-localization. CLSM data were confirmed by fluorescence cross correlation spectroscopy (FCCS) data, as will be shown in the corresponding section.

### 2.2. Flow Cytometry

FC is a technique for detection and measurement of the biological and physical characteristics of mainly cells and particles in suspension based on light scattering and fluorescence intensity emission. When focused light strikes particles or cells in a fluid sample, it gives out signals picked up by forward- and side-scatter light detectors. These signals are then converted for computer storage and data analysis and can provide information about various properties, such as the cells’ size and granularity. Optical filters in a FC can capture a range of wavelengths specific to fluorescent dyes. Fluorescent dyes that bind specifically to cellular constituents, such as DNA and proteins along with immunological detection of cell surface markers using antibody conjugated-fluorophores can indicate viable and dead cells [30,31]. FC also enables to investigate all aspects of apoptosis, from induction via surface receptors to DNA fragmentation in the late stages [32,33]. FC that uses impedance can detect necrosis without the need for fluorescent markers [34]. Therefore, FC is a widely used technique for studying cell toxicity, and also has been applied for studying the cellular response to nanomaterials.

**Figure 1:**
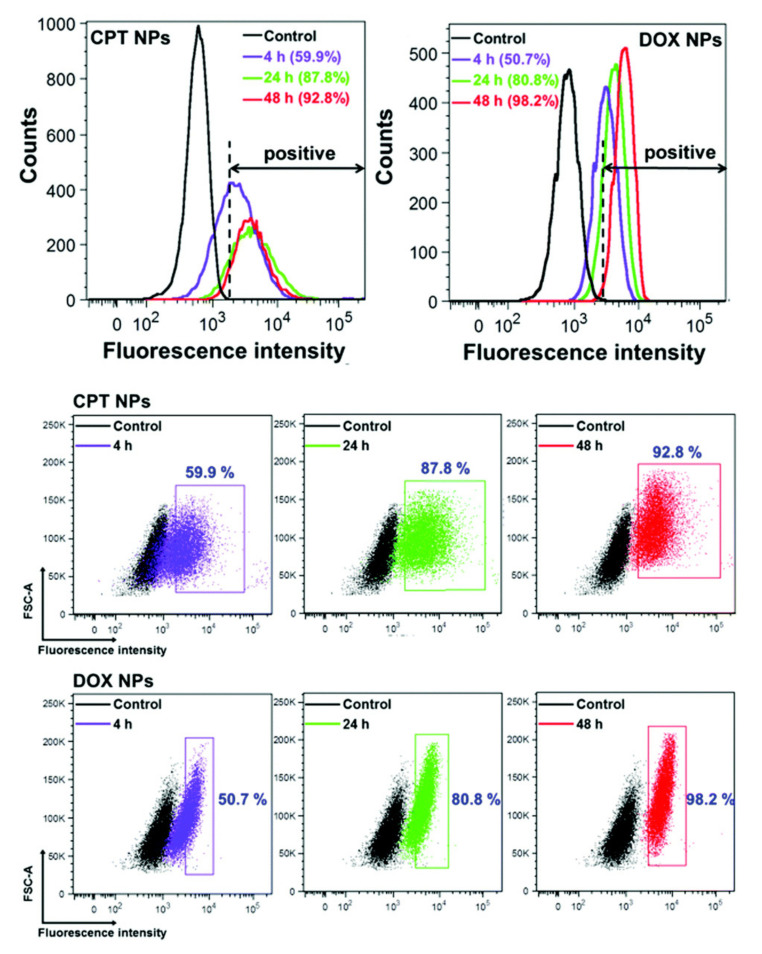
Flow cytometry analysis of cellular uptake of encapsulated anticancer drugs camptothecin (CPT NPs) and doxorubicin (DOX NPs) in HeLa cells after different incubation times. The control represents the unlabelled cells. The corresponding flow cytometry dot plots of HeLa cells after treatment with CPT NPs and DOX NPs for different times. Reproduced with permission from [5]. CC by 4.0.

FC is a high-throughput technique and allows the examination of tens of thousands of cells, providing statistically meaningful data and relative quantification of NP uptake inside a target cell [35,36]. FC data analysis, built upon the principle of gating, allows for the sorting of cell populations with nanomaterial uptake. When measuring NP uptake, it is essential to consider proper controls for gating parameters and threshold settings on forward- and side-scatter and fluorescent channels for detection. Fluorescence intensity histograms and two-parameter density plots are two ways of displaying this type of data (Figure 1). FC allows for identification of different cell subpopulations, quantifies the percentage of cells with NPs and without, and evaluates cell parameters such as granularity in parallel with nanomaterial uptake.

FC can track the changes in fluorescence per cell in a population as a function of time, quantifying the uptake kinetics [37]. The uptake of NPs begins with their initial adhesion to the cell surface and that subsequently, an energy dependent mechanism must be activated, which allows the internalization [38]. However, there are factors that hamper the cellular uptake process. A study by Yue et al. shows a critical rate-limiting step for NP internalization, which is the availability of cell surface area. In another case, NPs may attach but do not penetrate [39]. A computational study of cellular entry pathways shows NPs can be attached to the cells’ inner surface or stuck within the cell membrane [40]. Such scenarios would read out as false positives for actual uptake of NPs by FC. [41].

FC can provide information on the amount of NCs taken up by cells, mainly by recording fluorescence coming from the labelled nanomaterials, and in some cases by recording the increase in scattering from cells. FC can detect cells with fluorescently labelled NCs contained on their surface or within them. A priori, the technique is not the best to discriminate from NCs that are internalized or on the surface, for which microscopy measurements are better suited. However, it is possible as long as stringent sample preparation and proper controls are included. There are several steps to be taken before FC analysis, including adequate removal of all cell culture media and washing the treated cells with PBS, with cell fixation to preserve samples [38]. Treatment of cells at lower temperatures to block NP uptake processes also creates an experimental control condition for distinction between associated and internalized particles [35].

Once NPs are inside cells, FC can monitor their degradation kinetics. Romero et al. attached rhodamine (RhdB) to PLGA molecules to reliably track their degradation [42]. The method uses the fluorescence emission intensity of RhdB, which increases under the acidic environments found in lysosomes. As the PLGA NPs degrade, RhdB present in the hydrophobic core of the NPs is exposed to the endosomal pH, and this will increase the RhdB fluorescence inside the cell, reflecting the degree of exposure of the dye attached to the polymer to the endosome and the degree of PLGA degradation.

In a different study, Romero et al. studied the cell uptake of fluorescein-labelled PLGA NPs with encapsulated doxorubicin (DOX) in HepG2 cells, monitoring simultaneously the fluorescence from the NPs and from DOX [43]. According to the fluorescence intensity per cell and cellular uptake measurements, the amount of signal from the PLGA NPs and DOX rapidly increases during the first hours of incubation. Subsequently, the signal ratio increases as the fluorescence intensity of the fluorescein remains stable and the DOX signal inside the cell begins to gradually decrease after 24 h. One explanation for the disappearance of DOX is its capacity to stabilize the topoisomerase II complex in DNA, quenching its own fluorescence. It could also be that the PLGA NPs were progressively releasing DOX in the media and the uptake of non-encapsulated DOX follows different kinetics. Exocytosis could play a role in removing DOX after being released from PLGA NPs inside the cell. This work hints at the risks of following uptake of NPs by measuring the fluorescence of the encapsulated molecules that could be released during the uptake or intracellularly, which may lead to a false conclusion of the actual NP uptake. It could also be that PLGA NPs were progressively releasing DOX in the media and the uptake of non-encapsulated DOX follows a different kinetics. DOX could also be partially liberated through exocytosis after being released from PLGA NPs inside the cell. This work hints at the risks of following uptake of NPs by measuring the fluorescence of encapsulated molecules that could be released during the uptake or intracellularly, which may lead to a false conclusion of the actual NP uptake.

Imaging flow cytometers combine the speed, sensitivity, and abilities of flow cytometry with the detailed imagery and functional insights of microscopy [44]. These capabilities allow for the quantification of a fluorescent probes’ intensity and location on, in, or between cells. A study on micelle-mediated delivery used FC-imaging shows the uptake of siRNA into the cell cytoplasm [45]. The level of detail provided by such systems can track the internalization of nanomaterials/NPs and drug molecules in rare sub-populations and highly heterogeneous samples. Therefore, FC, coupled with imaging, is state-of-the-art for detecting NC internalization and drug release in live cells.

### 2.3. Förster Resonance Energy Transfer

The FRET technique is a fluorescence-based approach to study the interaction of molecules. The basis for FRET is a long range non radiative energy transfer between two light-sensitive molecules, one which is an excited fluorophore (the donor) and the second a fluorophore (the acceptor) that is excitable by the energy emitted from the donor. In order that FRET can occur, it requires a donor with an emission spectrum that partially coincides with the acceptor absorption spectrum. The donor/acceptor pair must be sufficiently close to each other (usually between 1 to 10 nm). Increasing the distance between the donor and acceptor results in a progressive decrease in transfer.

FRET can be described as:

D* + A → D + A*(1)

where the * denotes an electronically excited state. The rate constant of the energy transfer, *k_FRET_*, is a simple function of the distance in between the pair, *R*.

(2) $$kFRET=\frac{1}{\tau_{D}}{\left(\frac{R0}{R}\right)}^{6}$$

Here, *τ_D_* is the excited state lifetime of D in absence of *A* and *R*_0_ is the critical distance, i.e., the D-A distance at which the FRET efficiency is 50%.

The FRET efficiency is defined as the quantum yield of the process:

(3) $$E\equiv \frac{kFRET}{kFRET+\sum_{}^{}k_{i}}$$

where the sum is over all other decay processes but FRET from the excited state of D.

This dependence of FRET on distance, a molecular ruler, has been extensively used in biology and chemistry to study molecular interactions and to determine active sites in macromolecules. FRET can also be used to trace both degradation of carriers and the release of encapsulated drugs. For this, the drug must be fluorescent and the NCs labelled with another dye that can act as an acceptor or donor for the encapsulated drug. The attachment of fluorescent dyes to nanomaterials is a prerequisite for their monitoring by FRET [46]. The changes in the energy transfer between two fluorescent dyes can provide information about the intactness of NCs, their proximity inside the cell, and drug release, based on the dependence of FRET on distance. CLSM is the preferred technique to visualize FRET between different entities, including nanomaterials and the cargo, the core and coating of NCs, and the interaction between cellular components. To assess NC degradation, the donor and acceptor dyes must be associated to the molecular components of the NC. As the NC degrades, the transfer decreases as a result of the acceptor and donor moving further away from each other. FRET is also dependent on the number of donors and acceptors present, so losing one of them can already decrease FRET without actually meaning that the NC is degrading. Labelling conditions must be optimized for these types of experiments: too much or too less of a donor or an acceptor can result in an excess of fluorescence of one or the other and transfer may not be appreciated. FC can also measure changes in FRET. In the following, we highlight some examples of FRET in the characterization of NC delivery systems.

The design strategy of FRET donor and acceptor fluorophores are wide-ranging [47]. An important consideration is how fluorophores will withstand the cell culture medium, temperature, and time. As a recent study points out, the dye’s presence can affect the system’s physicochemical properties and vice versa [48]. Xiao et al. used FRET to distinguish how micelles release the cargo into the cell cytoplasm when they contact the cell membrane [49]. In that study, DAF inserted into the cell membrane, and Nile red dye inside the core of poly(ethylene glycol)-poly(lactic acid) micelles, were the donor/acceptor pair. These experiments elucidated that micelles effectively and quickly release a payload into the cytoplasm after contact with the cell membrane.

There are several ways to assess the stability of NPs using a dual fluorescence labelling strategy. DiI and DiD, two fluorophores able to interact by FRET, were encapsulated in the core as a way of monitoring the degradation of NPs [50]. DiI and DiD were loaded as the FRET donor/acceptor pair to show calcium alginate NCs deliver their contents inside cells once internalized [51]. This same strategy was applied to micellar NCs using a novel fluorescent dye pair [52]. Nuhn et al. used covalent dye labelling of separate batches of polymers followed by co-self-assembly into micellar NPs [53]. In this case, the fabrication of two fluorescent versions of a nanomaterial included the donor/acceptor pairs Cy3 and Cy5.

NC functions are tunable by substituting nanomaterials with different physicochemical properties. The successful application of NCs hinges upon their ability to carry cargo while resisting extracellular environments. After cellular uptake, intracellular conditions should degrade NCs and release the content. Thapaliya et al.’s study demonstrates the basics of an energy transfer scheme between cargo and NPs that simulates cargo release [54]. FRET provided information about the spatial association of the two dyes before and after uptake and consequently NC stability.

**Figure 2:**
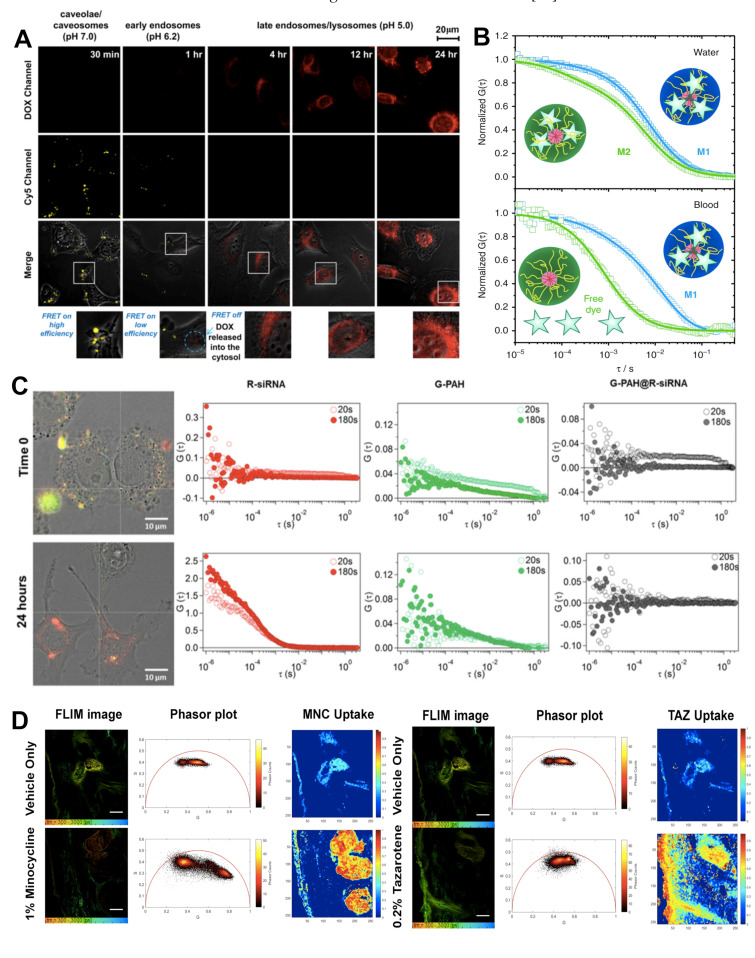
(**A**) Dual-emission fluorescence images of HT1080 cells; cells were incubated with DOX-loaded Cy5–NPCS nanoparticles for distinct durations and fluorescence images were then taken by CLSM in optical windows between 560 and 600 nm (DOX imaging channel) and 660 and 700 nm (Cy5 imaging channel) when irradiating NC suspensions at 488 nm. Reproduced with permission from [58]. Copyright 2011 Elsevier. (**B**) NIR-FCS studies of the loading stability of core-crosslinked micelle nanocarriers in blood. Normalized autocorrelation curves (symbols) and the corresponding fits (lines) are shown for core-crosslinked micelles that were either covalently (M1, blue colour) or noncovalently (M2, green colour) loaded with IRDye^®^800CW. Measurements in water (**upper panel**). The dye is mainly loaded to the core-crosslinked micelles and only a small fraction of free dye was detected for both systems. Measurements in the blood flow (velocity of 50 μL h^−1^) upon incubation with blood for 30 h (at 4 °C) (**lower panel**). The dye is fully released from M2, but still loaded to M1. Reproduced with permission from [75]. Copyright 2018 Springer Nature. (**C**) Representative cross-correlation experiment in live cells. A549 cells were imaged in transmission mode and cross-correlation was measured at specific locations inside the cell (white cross). Cells were incubated with the nanoparticles (N/P = 2) in RPMI complete medium. The cells were imaged just after incubation with the nanoparticles (Time 0), and after incubation for a further 24 h in complete medium under controlled conditions (37 °C and 5% CO_2_). The autocorrelation function in the red channel (ACF_R λ_ex_ = 633 nm, red markers) is associated with R-siRNA, and the autocorrelation function in the green channel (ACF_G λ_ex_ = 488 nm, green markers) is associated with G-PAH. The cross-correlation curve is represented by black markers (FCCS) and is associated with PAH/siRNA nanoparticles. Each location was measured in 20 runs of 10 s each. Average curves are reported after 20 s (runs 1–2) and 180 s (runs 3–20). (For interpretation of the references to colour in this figure legend, the reader is referred to the web version of this article.) Reproduced with permission from [29]. Copyright 2019 Elsevier. (**D**) Quantitative visualization of individual API uptake (MNC or TAZ) in the single component topical drug using the non-Euclidean phasor analysis algorithm. TSCPC-FLIM images of the ex vivo facial skin samples treated with BPX-05 containing no APIs (vehicle), and either 1% MNC or 0.2% TAZ were obtained from 500–550 nm with 780 nm two-photon excitation and 90 s acquisition time. The MNC (**left panel**) or TAZ (**right panel**) uptake images were computed using the non-Euclidean phasor analysis algorithm with two fluorescence references, including one endogenous (skin’s autofluorescence) and one exogenous (MNC or TAZ dried form, respectively). The scale bar is 100 μm. Reproduced with permission from [92]. Copyright 2020 Springer Nature.

FRET can take place between the labelling of the core and surface or particle and drugs. Some NCs are designed to degrade progressively as a result of hydrolysis. Drugs encapsulated in the core (hydrophobic) are liberated during core degradation, or through defects in the polymer matrix as is the case with PLGA [55]. Other types of NCs are susceptible to the cellular environment’s polarity, which mediates drug release. The release of hydrophobic molecules from polymer-based carriers into cell membranes was studied using FRET [56,57]. The degradation of NCs is traceable using the spectral properties of drugs. DOX is a commonly used drug in FRET studies with a fluorescence emission of an acceptor. Chen et al. used the FRET between Cy5 and DOX to track the disassembly of NPs in cells [58]. Cy5-NPCS NPs loaded with DOX have a FRET on a high-efficiency signal that finally disappears once DOX is released (Figure 2A). Spatial changes between NCs and their cargo are also measured using the autofluorescent anticancer drug agents, curcumin [59,60] and coumarin [61]. These works exemplify fluorescent labelling strategies for successful monitoring by FRET.

Wang and co-workers used tetraphenylethene-functionalized (TPE) (aggregation-induced emission luminogens, AIEgens) mesoporous silica NPs (FMSNs) as the FRET donor and encapsulated DOX as the FRET acceptor [62]. Emission spectrum of FMSNs (483 nm) overlaps with the absorption spectrum of DOX (480 nm), which enable the energy transfer between the FRET donor and acceptor. TPE emits strong blue fluorescence. NPs changed the colour from blue to red after encapsulation of DOX. Upon releasing the DOX, blue fluorescence was slowly recovered. The CLSM images of HeLa cells incubated with FMSNs indicated that the NCs were fast internalized inside the cells (1 h). The DOX was released in a more acidic environment inside cell endosomes or lysosomes and then transferred into the nucleus.

### 2.4. Fluorescence Correlation Spectroscopy/Fluorescence Cross-Correlation Spectroscopy

FCS is an advanced fluorescence microscopy technique based on recording temporal fluctuations of the fluorescence intensity [63,64]. It can monitor the motion of the fluorescently labelled molecules or NPs within an optically defined focal volume (typically less than 1 fL). Analysis of the recorded data yields information such as the translational diffusion coefficients, flow rates, chemical kinetic rate constants, rotational diffusion coefficients, molecular weights, and concentration [21,65,66,67,68,69,70]. FCS provides a suitable methodology for measuring the mobility, association, and ligand kinetics of biological molecules in the cellular environment.

FCS has been widely used to evaluate the adsorption of proteins onto, i.e., NP protein corona formation [71,72,73]. In a recent study, we looked at the influence of the surface coating on the intracellular behaviour of gold NPs (AuNPs) via FCS [65]. This study demonstrated that PEGylated AuNPs, in particular alkyl PEG600-coated AuNPs, which have a lower affinity to proteins, show a lower tendency to form aggregates intracellularly. In a similar vein, Eriksen et al. demonstrated the ex vivo diffusion dynamics of PEGylated liposomes [74]. The authors used PEGylated neutral, anionic, and cationic liposomes to measure the diffusion kinetics and biodistribution in a porcine eye.

FCS has also been used to monitor drug NCs in human blood using an NIR FCS setup [75]. In this study, the authors used NIR FCS to determine the loading stability of cross-linked polypeptide micellar NCs in blood (Figure 2B). The hydrodynamic radius of the micelles was first determined in water and later in flowing blood. Following a 30-h incubation period, they showed that there was a two-component diffusion profile, indicating the presence of free dye and micelle encapsulated dye, an important factor when determining the release of loaded drugs.

FCCS is a variant of FCS [76]. In this case, two lasers with different wavelengths excite a sample aiming to study the interaction of two species labelled with two different fluorophores [77]. The fluorophores must be spectroscopically different to minimize signal overlapping, cross excitation, or cross talk [78]. FCCS allows recording not only the autocorrelation function of the two species but also the correlation function between them. As such, it is possible to study species that are not distinguishable by their diffusive properties. The cross-correlation signal is highly sensitive, providing information on the interaction of small species, such as proteins in solution [79], although it is also possible to obtain information of particles inside the cells [80]. In the recent study by Di Silvio et al. [29], previously discussed in the section on CLSM, detailed information on the complexation of PAH and siRNA was obtained by FCCS. It shows complexation, formation, and colloidal stability of the complexes is dependent on the ratio of the charged amines in the PAH to the charged phosphate groups of the siRNA, while also showing a dependence on environmental factors such as pH and proteins present in media. Intracellularly, FCCS revealed that the PAH/siRNA complexes rearrange, forming large aggregates shortly after uptake before eventually disassembling and thus liberating the siRNA, which results in the loss of cross correlation between the two labelled species (Figure 2C). This was verified by the faster diffusion time of the liberated siRNA with respect to the complexed siRNA, which displays a much slower diffusion time.

### 2.5. Fluorescence Lifetime Imaging

The fluorescent lifetime of a molecule is defined as the average time it spends in the excited state before relaxation to the ground state via the emission of fluorescence [81,82]. The lifetime can range from picoseconds to hundreds of nanoseconds. This process is sensitive to the local micro-environment in which the fluorescent molecule is present. The fluorescent lifetime can be affected by changes in pH, changes in oxygen concentration, as well as the presence of other fluorescence-quenching species. As such, fluorescence lifetime imaging of inherently fluorescent or fluorescently labelled drugs/molecules can be easily traced via FLIM.

FLIM uses the fluorescent lifetime to provide contrast in a captured image rather than intensity. FLIM systems operate in one of two different domains, namely, the time domain or the frequency domain. Conceptually, time domain systems are easier to understand as they are direct measure of the fluorescence decay following pulsed excitation. Frequency domain systems rely on measuring the phase lag between the excitation, which is frequency modulated, and the fluorescence emission, as shown below. There are no differences in the lifetime values recorded using either the time or frequency domain.

As mentioned above, the fluorescent lifetime of a molecule can be affected by its local micro-environment. For example, changes in pH from one cellular environment to another can lead to changes in the fluorescent lifetime of a probe, as well as being useless for triggering the degradation of a delivery vessel [83]. Similarly, the fluorescent lifetime can also be affected by the polarity [84]. Temperature also influences the fluorescence lifetime of a probe, as demonstrated in a study by Okabe et al. in which the authors used the temperature-induced variation in the fluorescent lifetime as a type of fluorescent polymeric thermometer [85].

This sensitivity of the fluorescent lifetime to the local micro-environment can be taken advantage of to track the liberation of a fluorescent drug from a polymer NP, for example. If we look at the case of DOX, a widely used chemotherapeutic drug that happens to be intrinsically fluorescent, we can measure its fluorescent lifetime to determine whether or not it has been liberated from its delivery vessel [86,87,88,89,90]. In a recent study, it was demonstrated that the encapsulation of DOX inside PEO–PPO–PEO triblock copolymers, which self-assemble into micelles above the critical concentration, was possible. The encapsulation acts as a means to keep the DOX in an apolar environment, and as such we can distinguish it from free DOX via the different fluorescent lifetime, i.e., apolar vs. polar local micro-environment [90]. The lifetime value also distinguishes the location of DOX located inside the polar interior of the micelles or in the PEO corona. Lifetime measurements also provide a means to evaluate the breakdown of the polymer micelles, or lack thereof, as we can directly visualise the change in fluorescent lifetime from apolar to polar environments.

In these in vitro studies, the fluorescent lifetime of DOX inside polymer capsules is first measured. Following cellular uptake of the DOX-loaded capsules, the fluorescent lifetime is again measured to verify its release. The liberation process is triggered by the change in pH from media to the endosomal environment. Once the DOX is released, it intercalates which leads to a change in the fluorescent lifetime of DOX and serves as proof of its release from the vessel. Similarly, two-photon FLIM was used by Ge et al. [91], to monitor the delivery of coumarin-tagged polysaccharides in vitro. As in the case of DOX, the delivery of the tagged polysaccharides results in a change in the fluorescent lifetime of coumarin.

In another study, Jeong et al. demonstrated the use of two-photon FLIM with phasor analysis to visualize multicomponent drug distribution with human skin, i.e., ex vivo [92]. This not only highlighted the use of FLIM for studying drug distribution, but also the possibility of using two-photon FLIM to study drug distributions at greater depths while excluding cellular autofluorescence via phasor analysis. To do this, the authors formulated a topical gel solution containing minocycline (MNC) and tazarotene (TAZ), which have distinct fluorescence lifetimes from the lifetime of the skin’s autofluorescence. Using phasor analysis, they were able to demonstrate the local distributions and permeation of these drugs in the skin (Figure 2D). A similar ex vivo study by Alex et al. also demonstrates the power of FLIM for visualising the distribution and permeation of experimental anti-inflammatory drug formulations based on the change in fluorescence lifetime of the drugs in different environments [93].

## 3. Confocal Raman Microscopy

Raman spectroscopy is based on the inelastic scattering of light upon interaction with molecules [94], which provokes the vibration of chemical bonds resulting in specific energy shifts in the back-scattered light. The Raman spectrum is a chemical fingerprint of a molecule. Raman scattering is a weak effect, where most of the absorbed light by the molecule is elastically scattered at the same energy at which it was absorbed, a process called Rayleigh scattering. Only 1 in every 10^8^ molecules are inelastically scattered. Raman scattering is differentiated as Stokes scattering, observed when the photon transfers the energy to the molecule, or anti-Stokes scattering, when the molecule transfers energy to the photon. In spontaneous Raman spectroscopy, the sample is excited by the pump laser, and the inelastically scattered photons are detected in Stokes shifts [94]. Raman spectroscopy can be used for visualization of nanomaterials and their molecular components. Moreover, from the stretching and bending of excited bonds, information on the nanomaterial’s structure, shape, size, and surface properties can be obtained, which makes Raman spectroscopy a unique technique for studying the influence of the environment for nanomaterials in biological matrixes.

Raman spectroscopy can be successfully used to study the degradation of nano/micro particles. Li et al. took advantage of Raman spectroscopy to study in detail the degradation mechanism of MIL-100(Fe) nano- and microparticles, a type of MOF particle [95]. Raman spectroscopy was used to monitor changes in the microMOFs after incubation in PBS, as well to study the composition of different regions in the same particle. The degradation process of the microMOFs was slower in PBS than nanoMOFs, where particles degraded after 8 days. The degradation was observed to start in the outer surface of MOFs, as formation of a grey-coloured shell around a red-coloured core. While the Raman spectrum in the core corresponds to the characteristic peaks of non-degraded MOFs, the shell area exhibits only a broad peak, observed in degraded, amorphous sample. The mechanism of the degradation was related to the progressive replacement of the trimesate ligands from the MOF structure by phosphate ions present in high amounts in PBS. Moreover, Raman spectroscopy was used to prove that the encapsulation of the anticancer drug, Gemcitabine monophosphate, or coating of the external surface of the NP with cyclodextrin-phosphate, did not lead to degradation of the MOFs.

In confocal Raman microscopy (CRM), a Raman laser is interfaced to an optical microscope. CRM has been used to image cell uptake of NPs in a label-free manner [95]. One of the advantages of CRM that it does not requires cell fixation, and cells can be kept alive. Klein et al. have demonstrated the advantages of the CRM compared to the immunofluorescence (IF) staining [96]. The authors demonstrated that both methods allow monitoring the nucleus, cytoskeleton, and Golgi apparatus of the cells. The information from both techniques overlaps; however, only CRM can give the precise information of the uptake of NPs in cells without labelling or any other modification. It can also be applied to trace the distribution of NPs in living cells, as well as for investigating drug delivery inside cells.

CRM allows studying the intracellular degradation of NPs. In a pioneering work, Apeldoorn et al. successfully demonstrated the degradation of PLGA microspheres inside of macrophages [97]. The internalized microspheres showed signs of degradation after 1 week, while PLGA without presence of macrophages did not show any signs of degradation. The degradation of the PLGA NPs can be monitored by comparison of the two bands representing the C–COO stretch of lactic acid (875 cm^−1^) and C=O vibration of the ester group (1768 cm^−1^). Once degradation occurred, there was a decrease in the intensity at the 1768 cm^−1^ band, due to the hydrolysis and reduction of the number of the ester bonds in the PLGA. However, the hydrolysis did not affect the intensity of the band at 875 cm^−1^. We mention here again, the study by Romero et al. which used CRM to study intracellular degradation of the PLGA NPs in PBS and HepG2 cell [42]. The authors demonstrated that the degradation rate depends on the copolymer composition by comparing the NPs with 15% and 35% glycolide (PLGA15 NPs and PLGA35 NPs, respectively). PLGA15 NPs exhibits the signs of degradation, while the PLGA35 NPs did not show the change in intensity of the bands. Therefore, it was concluded that the PLGA35 NPs, which contain higher amounts of glycolide, degraded more slowly than the PLGA15 NPs.

Cherneko et al. used Raman spectroscopy with optical microscopy to track the intracellular delivery and the degradation of PLGA and polymers-poly(ε-caprolactone) (PCL) NPs in cervical carcinoma cell lines (HeLa cells) [98]. PCL NPs were quickly taken up into the cells (7 h of incubation). The degradation of the PCL NPs was monitored at two time points (7 h and 17 h) after NP uptake by the cells was stopped. The degradation occurred via hydrolysis of the ester bonds, corresponding to a decrease in the 1735 cm^−1^ band. In-depth analysis of the NPs’ distribution in the cells showed that the NPs tended to aggregate after entering the cells and were randomly distributed in the cytoplasm. Lipid-rich inclusions appeared along NP aggregations. These could be observed as subcellular vesicle formations, showed in appearance of the phospholipid-character peaks in the C–H stretching region (2920 cm^−1^) and the ester linkages region (1745 cm^−1^). The NPs, after a longer time of degradation, were enwrapped in Golgi-associated vesicles of the late endosomes. Later, peaks specific to PCL disappeared, while the phospholipid inclusions were still present, before samples were fully degraded. Compared to PCL, the PLGA NPs had faster degradation kinetics. NPs were endocytosed after 2 h of incubation in HeLa cells. Degradation of PLGA NPs occurred in a similar process to PCL NPs after 3 h from stopping the NP uptake.

Tolstik et al. also took advantage of CRM to investigate the uptake and degradation of porous silicon NPs (SiNPs) with or without photoluminescence (PL) in breast cancer cells (MCF-7 cell line) [99]. After 6 h and 9 h of incubation, NPs were detected mostly on the cell membrane, and then after 24 h of incubation the NPs were localized in the cell cytoplasm. Changes in NPs inside cells were then investigated for two weeks. Once the NPs were detected in the cell cytoplasm, the band corresponding to the nanocrystalline silicon present in the cells (520 cm^−1^) was shifted to lower wavenumbers (516 cm^−1^) and broadens, hinting at a partial degradation of the SiNPs. Moreover, a signal typical for the amorphous silicon (480 cm^−1^) could be recorded. After longer incubation times (13 h), a significant decrease in the signal together with a further shift to 513 cm^−1^ appeared together with the growth of the amorphous phase. CRM has been used to track the uptake and internalization of Laponite NPs, a synthetic layered clay, into the J774 macrophage cell line [100]. Laponite NPs were functionalized with serum proteins from foetal bovine serum (FBS). After 24 h of incubation, the NPs were found inside the cytosol of the cells.

Raman spectroscopy is also valuable to monitor the intracellular release of drugs in living cells. Lee et al. investigated the drug release (DOX) from surface-capped MSNs using label-free in situ Raman monitoring in human embryonic kidney 293 (HEK293) cells and human renal carcinoma (A498) cells [101]. NPs were first functionalized with amine groups by 3-aminopropyl triethoxysilane (APTES) (APTES-MSNs), and then were further modified by AuNPs or BSA (denoted as GNP-MSNs and BSA-MSNs, respectively). The release of DOX from GNP-dMSN was investigated at pH 5 and pH 7. The observation of the main Raman peak of DOX (454 cm^−1^) gave the information about the release process from the NPs. At pH 5, the Raman peaks for DOX increased in intensity, while at pH 7 no increase in intensity was observed. This indicates that DOX was released in large amounts only at a more acidic pH. Moreover, the cellular distribution of the delivered drugs via Raman mapping (intensity at 454 cm^−1^) was observed. After short incubation times (6 h) of the DOX-loaded capped-MSNs in A498 cells, the DOX signal in the cells increased significantly, while after incubation in HEK293 cells, the DOX signal increased slightly only after 24 h of incubation. This indicated that DOX was preferentially released faster in the more acidic cancer environment.

Surface-enhanced Raman scattering (SERS) is a technique for enhancing the intensity of weak Raman bands. SERS is based on the electromagnetic enhancement of Raman signals by the interaction of molecules with a metallic surface [102]. SERS spectroscopy was used to monitor the controlled release of the anti-cancer drugs azacitidine (AZA) and decitabine (DAC) from AuNPs modified with mercaptobenzoic acid (MBA) and with folic acid bound to para-aminothiophenol (PATP) in the shell of AuNPs [103]. Release of both drugs in buffer solution was fast at a low pH (pH 4 and 5.5), while no release was observed in pH 7.4. A strong Raman signal was enabled due to the presence of an MBA linker, which is a well-known Raman marker. While drugs were immobilized on the NPs, the signal was reduced due to the shielding effect. However, once the drug was released, the SERS signal increased again. Santiago et al. also took advantage of SERS to investigate the targeted delivery and controlled release of gemcitabine (GEM) from NCs [104]. The authors covalently modified AuNPs with thiol linkers and immobilized GEM on the NPs through a pH-sensitive amine bond. They further modified NPs with folic acid or transferrin (TF) for optimal targeting. In a similar strategy to the previously described work, AuNPs were modified with MBA. Kurzątkowska et al. used a plasmonic NC-based SERS nanogrid sensor to investigate the targeted delivery and control release of anticancer drugs (gemcitabine) [105]. The authors used the SERS sensor modified with AuNPs with immobilized gemcitabine and folic acid. GEM was released only in more acidic conditions, corresponding to a cancer-cell environment. GEM release was induced by the presence of glutathione, which is located in high concentrations in many types of cancer cells.

## 4. In Vivo Techniques

### 4.1. Fluorescence Imaging

One of the biggest drawbacks to in vivo fluorescence imaging is cellular autofluorescence. However, with the advent of fluorescent probes that emit in the near-infrared (700–2000 nm), the so-called second biological window, there has been renewed interest in its use [106,107,108]. Such is the penetration depth in this optical window that applications can range from monitoring the biodistribution of NPs to performing liver tumour surgery to in vivo information storage and decoding [109,110,111]. However, more traditional applications are still the most common application of in vivo fluorescence imaging.

Li et al. used thermally activated delayed fluorescence (TADF) organic dots to realise time-resolved and confocal imaging in live cells and in vivo [112]. These organic dots consisted of the TADF fluorophore 2,3,5,6-tetracarbazole-4-cyano-pyridine (CPy), which display a long fluorescence lifetime of approximately 9.3 μs in water and are also very bright. The green emission of the organic dots, coupled with the long fluorescence lifetime, makes them easily distinguishable from autofluorescence. To demonstrate their effectiveness, the organic dots were first tested in HeLa cells and later applied to zebrafish models to obtain confocal and FLIM images.

Simultaneously, drug release and carrier degradation processes are trackable at multiple wavelengths using multispectral fluorescence imaging. Zhang and colleagues developed a dual fluorescent drug delivery system called DOX-loaded genipin-crosslinked globin and PEI NPs (Gb-G-PEI/DOX NPs), which included DOX as the fluorescent antitumour drug and Gb-G-PEI NPs as the self-fluorescent NC [113]. Mice bearing tumours were used as models and after the administration of the Gb-G-PEI/DOX NPs, the tumour therapy effect was monitored in real-time.

FRET has also been applied in vivo studies. The FRET imaging criteria for in vivo are much more demanding and challenging than those in vitro as it requires higher sensitivity to pass through thick biological barriers. A FRET pair must withstand dynamic changing environments. The strategy for incorporating fluorophores into the NCs is critical for the application. For example, inserting the dye inside the NC will make it easier to discriminate the NC’s integrity from fluorophore molecules that may have been detached using other less robust incorporation methods. 

**Figure 3:**
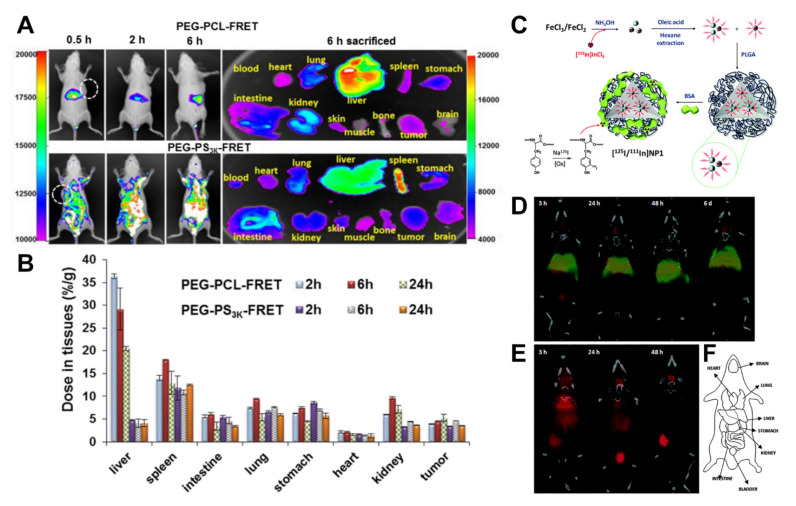
(**A**) In vivo behaviour of two different polymeric micelles (PEG-PCL and PEG-PS) by FRET. PEG-PCL with its PCL end conjugated with a FRET pair of Cy5 (donor) or Cy5.5 (acceptor). Mice were i.v. injected with PEG-PCL-FRET at 10 mg/kg and imaged at 0.5, 2, and 6 h and then sacrificed and dissected. Reproduced with permission from [114]. Copyright 2018 American Chemical Society. (**B**) The polymer concentration in each tissue was analysed. Data are reported as the mean (SD) for triplicate samples. The imaging was taken by the FRET fluorescence (Ex/Em = 640 nm/720 nm). Reproduced with permission from [114]. Copyright 2018 American Chemical Society. (**C**) Schematic of the preparation of dual-labelled [^125^I/^111^In]NP1 NPs. Reproduced with permission from [125]. Copyright 2015 Royal Society of Chemistry. (**D**) Coronal SPECT images of the biodistribution of [^125^I/^111^In]NP1 and (**E**) [^125^I]BSA after intravenous administration into mice; red colour: ^125^I; green colour: ^111^In. Images were co-registered with a CT image of the same animal to anatomically localize the SPECT signal. CT images were adjusted in the Y axis at each time point for appropriate fitting with the tracer distribution image. Reproduced with permission from [125]. Copyright 2015 Royal Society of Chemistry. (**F**) A schematic of the different organs. Reproduced with permission from [125]. Copyright 2015 Royal Society of Chemistry.

Micelles produced from PEG-block-PCL and PEG-block-poly(d,l-lactide) (PEG-PDLLA) were compared in vivo for how quickly and by what mechanism they were cleared [114]. Cy5 and Cy5.5 dyes attached to PEG molecules’ hydrophobic chain ends were used to fabricate FRET micelles for their subsequent tracing in mice (Figure 3A,B). Imaging taken by the FRET fluorescence showed these micelles could enter tumours and were very stable with cell membranes, but they eventually dissociated within cells. FRET can also be applied for monitoring the delivery of drugs from NCs in tumours, and simultaneously monitored by in vivo fluorescence imaging [115,116]. The incorporation of FRET in a photodynamic therapy (PDT) paradigm was used to track NC performance and confirm the delivery of photosensitizing agents in real time [117].

### 4.2. Nuclear Imaging: Positron Emission Tomography and Single-Photon Emission Computed Tomography

PET and SPECT are two types of nuclear imaging based on recording beta and gamma emissions, respectively, which can assess the biodistribution of the NCs and payloads in animal models, as well as their biological fate. Both techniques are minimally or non-invasive and require the administration of radiotracers to generate the signal. PET/SPECT can also be applied for the study of the stability and degradation of radiolabelled NCs in animals using proper labelling strategies [118]. PET and SPECT are entering clinical use with potential applications for monitoring drug release. Several recent studies have shown that liposomes containing DOX are taken up and retained in tumours, demonstrating their enhanced permeability and retention effect [119,120].

Radiolabelling selected cargo and the NCs are prerequisites for PET and SPECT. Mentioned below are some of the most common approaches. Mixing in trace amounts of the hot precursor with cold precursor during the NC preparation assembly prep is a simple way to generate radiolabelled NCs. There also is the physical entrapment of the radioactive cargo in the NCs and attachment of radiotracers cation exchange and chelation. For metal-based carriers, there is mixing in bulk metal with a small amount of the radionuclide. The variation on all these radiolabelling methods and the different available radiotracers has been extensively reviewed elsewhere [121,122,123].

In order to prevent the radiotracer’s detachment or leakage during harsh environments, surface functionalization, which allows for covalent bonding, is the most robust. In the following, we will discuss studies by our lab that used PET and SPECT for quantitative in vivo assessment and tracking of NPs. Detection of NPs using PET relies on the detection of positron-emitting radionuclides. In Pérez-Campaña et al., radiolabelling was performed by colliding protons into aluminium oxide NPs with sizes ranging from a few nanometres to micrometres, changing some of the ^16^O atoms to radioactive ^13^N atoms [124]. After incorporating the ^13^N atoms into the NPs, PET tracked their location in rats. The results from these experiments were able to show that the size of the NPs can influence their biodistribution and accumulation in different organs.

Multiple-isotope labels and their overlapping signals can help to visualize the NC degradation process; for example, the incorporation of positron or gamma emitters at the core or the shell of the NCs. An advantageous feature of the SPECT technique is that it can discriminate between multiple isotopes, provided that they have different spectral emission. Previously, we studied the degradation of PLGA-NPs in vivo by energy-discriminant SPECT [125]. The radiolabelling method combined encapsulation of ^111^In-doped iron oxide NPs in the core with a BSA surface coating containing ^125^I (Figure 3C). Notably, ^125^I and ^111^In do not have coincident emission bands. SPECT resolved each radioisotope independently after intravenous administration in rats. The labelling of the carrier and core with different radionuclides makes possible the NCs’ stability and dynamics, determining the fate of the core and coating. The two radionuclides co-localizing shows that while the PLGA core accumulated in the liver and lungs and were relatively stable for long periods, the BSA coating degrades and is progressively eliminated by the bladder (Figure 3D–F).

Bindini et al. have recently reported a study of the in vivo degradation of mesoporous NPs coated with PEG by PET imaging [126]. Mesoporous silica hydrolyses at a neutral and basic pH, which leads to its dissolution. Its degradation has been extensively studied in in vitro simulating in vivo conditions. However, in vivo it becomes rather difficult to differentiate between the actual silica NP and the products that come from its degradation for fate studies. The authors prepared core–shell silica NPs with a dense silica core and a mesoporous silica shell and labelled either one or the other with low quantities of ^89^Zr, a positron emitter suitable for PET studies. The NPs were the same in both cases, just differing in the location of the label. Because of this, when the distribution patterns in the mice models for both situations were compared following intravenous administration, conclusions about the degradation of the mesoporous silica shell could be obtained. When ^89^Zr is trapped in the hard core of the NPs, it is observed that NPs accumulate in the liver and the biodistribution does not change for the first 6 h. When the label is placed in the shell, a rapid release of the radioisotope is observed, with partial excretion already in the first two hours post injection, followed by a slower accumulation in the bones. The fast degradation kinetics in vivo can be extrapolated.

### 4.3. In Vitro vs. In Vivo Studies

In vivo studies, though more complex than in vitro ones, demand techniques not so accessible such as PET/SPECT, but provide a more “realistic” picture of the fate of the NCs and encapsulated drugs, which has a direct impact on the evaluation of the therapeutic efficacy of the NCs and their clinical relevance. In vitro studies on the other hand are more accessible in terms of availability of techniques, which are usually fluorescence based, and provide a useful pre-screening evaluation of the potential of NCs to deliver drugs in cells and tissues, and bring insight into the fate of NCs and drugs at the cellular level, which is highly complementary to in vivo studies [127]. The advantages and disadvantages of the different in vitro and in vivo techniques presented in this manuscript have been summarized in Table 1, highlighting which information can be obtained from each technique for the assessment of the fate of NCs and NC/drug conjugates, which we believe also hints at the importance of combining in vitro and in vivo studies.

## 5. Summary and Perspectives

We have reviewed here different techniques that are employed for tracing the fate of NCs for drug delivery, focusing on studies of the degradation of NCs and release of encapsulated drugs. Both aspects are often correlated as in many cases encapsulated drugs can only be delivered if the carrier degrades or breaks. We have seen that most of the techniques used for these studies require the labelling of the carriers, mainly with fluorescence tags but also with radio and Raman tags. There is not one technique that can be used unequivocally for studying drug release or NC degradation in biological matrixes and each nanomaterial formulation must be considered individually regarding the choice of a technique and labelling strategy. Raman provides a means of tracing the fates of NCs and drugs without labelling, relying on the intrinsic Raman bands of the nanomaterials and drugs, when these are present and can be distinguished from Raman bands coming from the biological environment. CLSM and FC offer the means to trace variations in the fluorescence between labelled NCs and drugs or between two components in the NC that can correlate with the release of drugs or the disassembly of the carrier, based on the dependence of FRET on the distance between the species participating in the transfer. FLIM imaging allows recording changes in the environment of a dye, such as polarity, pH, and ionic strength, which can be used for the release of a fluorescent molecule from a carrier to the surrounding biological environment. FCS and FCCS are spectroscopic techniques that allow to trace the temporal fluctuations of fluorescence and between two different fluorescence species in a confocal volume. From changes in the autocorrelation and cross correlation curves, the release of fluorescent molecules from a carrier, the degradation of molecules or carriers to smaller sizes, or the separation of two fluorescent species (as in FRET) can be inferred.

In vivo fluorescence imaging of the carriers and drug is possible, requiring dyes that can be distinguished from the cell autofluorescence. Nuclear imaging techniques (PET, SPECT), more quantitative than fluorescence, allow for measuring the time evolution of the distribution of the radiolabelled carriers or drugs per organ through measuring activity, which can be correlated with the dose of the materials administrated. Both fluorescence and nuclear imaging have been applied to trace NC stability through multiple labelling strategies, i.e., dual core and coating labelling, and labelling of several components.

Fate studies that correlate with changes in the NCs, such as their degradation, loss of components and drug, or therapeutic delivery, are fundamental for the evaluation of the potential of a nanoformulation to be effective in delivering a drug or therapy and necessary for the translation of nanomedicines, especially in vivo. Strategies for tracing fate often require complex labelling procedures and a combination of techniques that are not available in every laboratory, such as PET/SPECT. However, in the future of nanotechnologies in medicine, we believe this will play a fundamental role.

## Figures and Tables

**Table 1:** Summary of the techniques presented, highlighting their advantages and disadvantages for fate studies in vitro and in vivo.

Techniques	Advantages	Disadvantages	Experimental Type	References
FC	Quantitative measurements of NC/drug uptake in a cell population measuring fluorescence at single cell level.	Requires fluorescence labelling. Cannot discriminate easily between NPs. inside or on the surface of cells.	In vitro	[34,35,36,37,47]
CLSM	Able to visualize the location of NCs and drugs inside cell organelles.	Requires fluorescence labelling. Can give false results due to detached dyes from NCs.	Mainly in vitro	[23,24,25,26,27]
FRET	Can determine the proximity of two fluorescence molecules in vitro and in vivo. can be used for studying release of drugs or degradation of NPs	Requires labelling and sometimes complex. Molecular design.	In vitro/in vivo	[50,51]
FCS/FCCS	Allows for studying diffusion of fluorescent molecules and the temporally correlate the association of labelled species. Can be applied for studying degradation of NCs or Drug release.	Requires fluorescence labelling. Photobleaching can difficult measurements	In vitro	[63,64,79,80]
FLIM	Imaging is based on measuring lifetime of fluorescence molecules that can be traced even with low intensity. Lifetime measurements are sensitive to environmental conditions such as pH, ionic strength, temperature.	Requires fluorescence labelling. Can give false results due to detached dyes from NCs.	In vitro/in vivo	[81,82,83,84,85]
Fluorescence Imaging	Gives precise information about NCs/drug distribution biodistribution (in vivo).	Long exposure to fluorescent light can cause bleaching. Dye detachment can lead to a false localization of NPs or drugs. Not quantitative.	In vivo	[106,107,108]
Raman	Minimal sample preparation. Non-invasive. Non-destructive. Label-free manner visualization of NPs and drug in cells and tissues. Co localization studies of NPs and drug without additional labelling.	Limited confocality. Time consuming. Fluorescence can interfere with measurements.	In vitro	[94,96]
PET/SPECT	Based on the detection of radioactive element. Quantitative. In vivo biodistribution of NPs/drugs can be quantitatively determined.	Requires radiolabelling nanomaterials. Can give false. Information if radioisotopes detach. Molecules and imaging techniques are not easily accessible.	In vivo	[118,119,120,121,122,123]

## Data Availability

Not applicable.

## References

[1] Min Y., Caster J.M., Eblan M.J., Wang A.Z. (2015). Clinical Translation of Nanomedicine. Chem. Rev..

[2] Kwon G.S. (1998). Diblock Copolymer Nanoparticles for Drug Delivery. Crit. Rev. Ther. Drug Carrier Syst..

[3] Reibetanz U., Claus C., Typlt E., Hofmann J., Donath E. (2006). Defoliation and Plasmid Delivery with Layer-by-Layer Coated Colloids. Macromol. Biosci..

[4] Simón-Gracia L., Hunt H., Scodeller P., Gaitzsch J., Kotamraju V.R., Sugahara K.N., Tammik O., Ruoslahti E., Battaglia G., Teesalu T. (2016). IRGD Peptide Conjugation Potentiates Intraperitoneal Tumor Delivery of Paclitaxel with Polymersomes. Biomaterials.

[5] Xu Z., Liu S., Kang Y., Wang M. (2015). Glutathione- and PH-Responsive Nonporous Silica Prodrug Nanoparticles for Controlled Release and Cancer Therapy. Nanoscale.

[6] Jin S., Ye K. (2007). Nanoparticle-Mediated Drug Delivery and Gene Therapy. Biotechnol. Prog..

[7] Gregori M., Masserini M., Mancini S. (2015). Nanomedicine for the Treatment of Alzheimer’s Disease. Nanomedicine.

[8] Pelaz B., Alexiou C., Alvarez-Puebla R.A., Alves F., Andrews A.M., Ashraf S., Balogh L.P., Ballerini L., Bestetti A., Brendel C. (2017). Diverse Applications of Nanomedicine. ACS Nano.

[9] Kumari A., Yadav S.K., Yadav S.C. (2010). Biodegradable Polymeric Nanoparticles Based Drug Delivery Systems. Colloids Surf. B Biointerfaces.

[10] Battaglia L., Gallarate M. (2012). Lipid Nanoparticles: State of the Art, New Preparation Methods and Challenges in Drug Delivery. Expert Opin. Drug Deliv..

[11] Porter C.J.H., Trevaskis N.L., Charman W.N. (2007). Lipids and Lipid-Based Formulations: Optimizing the Oral Delivery of Lipophilic Drugs. Nat. Rev. Drug Discov..

[12] Liong M., Lu J., Kovochich M., Xia T., Ruehm S.G., Nel A.E., Tamanoi F., Zink J.I. (2008). Multifunctional Inorganic Nanoparticles for Imaging, Targeting, and Drug Delivery. ACS Nano.

[13] De la Fuente J.M., Grazu V. (2012). Nanobiotechnology: Inorganic Nanoparticles vs Organic Nanoparticles.

[14] Wang L., Zheng M., Xie Z. (2018). Nanoscale Metal–Organic Frameworks for Drug Delivery: A Conventional Platform with New Promise. J. Mater. Chem. B.

[15] Bulbake U., Doppalapudi S., Kommineni N., Khan W. (2017). Liposomal Formulations in Clinical Use: An Updated Review. Pharmaceutics.

[16] Chan J.M., Valencia P.M., Zhang L., Langer R., Farokhzad O.C. (2010). Polymeric Nanoparticles for Drug Delivery. Methods Mol. Biol. Clifton NJ.

[17] Graybug Vision a Depot Formulation of Sunitinib Malate (GB-102) in Subjects with Neovascular (Wet) Age-Related Macular Degeneration. https://clinicaltrials.gov/ct2/show/NCT03249740?term=sunitinib.

[18] Jahanshahi M., Babaei Z. (2008). Protein Nanoparticle: A Unique System as Drug Delivery Vehicles. Afr. J. Biotechnol..

[19] Coustet M., Irigoyen J., Garcia T.A., Murray R.A., Romero G., Susana Cortizo M., Knoll W., Azzaroni O., Moya S.E. (2014). Layer-by-Layer Assembly of Polymersomes and Polyelectrolytes on Planar Surfaces and Microsized Colloidal Particles. J. Colloid Interface Sci..

[20] Li S.-D., Huang L. (2010). Stealth Nanoparticles: High Density but Sheddable PEG Is a Key for Tumor Targeting. J. Controlled Release.

[21] Murray R.A., Qiu Y., Chiodo F., Marradi M., Penadés S., Moya S.E. (2014). A Quantitative Study of the Intracellular Dynamics of Fluorescently Labelled Glyco-Gold Nanoparticles via Fluorescence Correlation Spectroscopy. Small.

[22] Romero G., Estrela-Lopis I., Zhou J., Rojas E., Franco A., Espinel C.S., Fernández A.G., Gao C., Donath E., Moya S.E. (2010). Surface Engineered Poly(Lactide-Co-Glycolide) Nanoparticles for Intracellular Delivery: Uptake and Cytotoxicity—A Confocal Raman Microscopic Study. Biomacromolecules.

[23] Romero G., Rojas E., Estrela-Lopis I., Donath E., Moya S. (2011). Spontaneous Confocal Raman Microscopy—A Tool to Study the Uptake of Nanoparticles and Carbon Nanotubes into Cells. Nanoscale Res. Lett..

[24] Huang F., Watson E., Dempsey C., Suh J., Weissig V., Elbayoumi T., Olsen M. (2013). Real-Time Particle Tracking for Studying Intracellular Trafficking of Pharmaceutical Nanocarriers. Cellular and Subcellular Nanotechnology.

[25] Gibbs-Flournoy E.A., Bromberg P.A., Hofer T.P., Samet J.M., Zucker R.M. (2011). Darkfield-Confocal Microscopy Detection of Nanoscale Particle Internalization by Human Lung Cells. Part. Fibre Toxicol..

[26] Shang L., Nienhaus K., Jiang X., Yang L., Landfester K., Mailänder V., Simmet T., Nienhaus G.U. (2014). Nanoparticle Interactions with Live Cells: Quantitative Fluorescence Microscopy of Nanoparticle Size Effects. Beilstein J. Nanotechnol..

[27] Murray R.A., Escobar A., Bastús N.G., Andreozzi P., Puntes V., Moya S.E. (2018). Fluorescently Labelled Nanomaterials in Nanosafety Research: Practical Advice to Avoid Artefacts and Trace Unbound Dye. NanoImpact.

[28] Smith S.A., Selby L.I., Johnston A.P.R., Such G.K. (2019). The Endosomal Escape of Nanoparticles: Toward More Efficient Cellular Delivery. Bioconjug. Chem..

[29] Di Silvio D., Martínez-Moro M., Salvador C., de los Angeles Ramirez M., Caceres-Velez P.R., Ortore M.G., Dupin D., Andreozzi P., Moya S.E. (2019). Self-Assembly of Poly(Allylamine)/SiRNA Nanoparticles, Their Intracellular Fate and SiRNA Delivery. J. Colloid Interface Sci..

[30] Eustaquio T., Leary J.F., Reineke J. (2012). Single-Cell Nanotoxicity Assays of Superparamagnetic Iron Oxide Nanoparticles. Nanotoxicity.

[31] García-Rodríguez A., Kazantseva L., Vila L., Rubio L., Velázquez A., Ramírez M.J., Marcos R., Hernández A. (2019). Micronuclei Detection by Flow Cytometry as a High-Throughput Approach for the Genotoxicity Testing of Nanomaterials. Nanomaterials.

[32] Lanna E.G., Siqueira R.P., Machado M.G.C., de Souza A., Trindade I.C., Branquinho R.T., Mosqueira V.C.F. (2021). Lipid-Based Nanocarriers Co-Loaded with Artemether and Triglycerides of Docosahexaenoic Acid: Effects on Human Breast Cancer Cells. Biomed. Pharm..

[33] Guo W., Song Y., Song W., Liu Y., Liu Z., Zhang D., Tang Z., Bai O. (2020). Co-Delivery of Doxorubicin and Curcumin with Polypeptide Nanocarrier for Synergistic Lymphoma Therapy. Sci. Rep..

[34] Ostermann M., Sauter A., Xue Y., Birkeland E., Schoelermann J., Holst B., Cimpan M.R. (2020). Label-Free Impedance Flow Cytometry for Nanotoxicity Screening. Sci. Rep..

[35] Lesniak A., Salvati A., Santos-Martinez M.J., Radomski M.W., Dawson K.A., Åberg C. (2013). Nanoparticle Adhesion to the Cell Membrane and Its Effect on Nanoparticle Uptake Efficiency. J. Am. Chem. Soc..

[36] Salvati A., Nelissen I., Haase A., Åberg C., Moya S., Jacobs A., Alnasser F., Bewersdorff T., Deville S., Luch A. (2018). Quantitative Measurement of Nanoparticle Uptake by Flow Cytometry Illustrated by an Interlaboratory Comparison of the Uptake of Labelled Polystyrene Nanoparticles. NanoImpact.

[37] Shin H., Kwak M., Lee T.G., Lee J.Y. (2020). Quantifying the Level of Nanoparticle Uptake in Mammalian Cells Using Flow Cytometry. Nanoscale.

[38] Jochums A., Friehs E., Sambale F., Lavrentieva A., Bahnemann D., Scheper T. (2017). Revelation of Different Nanoparticle-Uptake Behavior in Two Standard Cell Lines NIH/3T3 and A549 by Flow Cytometry and Time-Lapse Imaging. Toxics.

[39] Yue T., Zhou H., Sun H., Li S., Zhang X., Cao D., Yi X., Yan B. (2019). Why Are Nanoparticles Trapped at Cell Junctions When the Cell Density Is High?. Nanoscale.

[40] Lin J., Miao L., Zhong G., Lin C.-H., Dargazangy R., Alexander-Katz A. (2020). Understanding the Synergistic Effect of Physicochemical Properties of Nanoparticles and Their Cellular Entry Pathways. Commun. Biol..

[41] Hou S., Sikora K.N., Tang R., Liu Y., Lee Y.-W., Kim S.T., Jiang Z., Vachet R.W., Rotello V.M. (2016). Quantitative Differentiation of Cell Surface-Bound and Internalized Cationic Gold Nanoparticles Using Mass Spectrometry. ACS Nano.

[42] Romero G., Echeverría M., Qiu Y., Murray R.A., Moya S.E. (2014). A Novel Approach to Monitor Intracellular Degradation Kinetics of Poly(Lactide-Co-Glycolide) Nanoparticles by Means of Flow Cytometry. J. Mater. Chem. B.

[43] Romero G., Murray R.A., Qiu Y., Sanz D., Moya S.E. (2013). Layer by Layer Surface Engineering of Poly (Lactide-Co-Glycolide) Nanoparticles: A Versatile Tool for Nanoparticle Engineering for Targeted Drug Delivery. Sci. China Chem..

[44] Han Y., Gu Y., Zhang A.C., Lo Y.-H. (2016). Review: Imaging Technologies for Flow Cytometry. Lab. Chip.

[45] Omedes Pujol M., Coleman D.J.L., Allen C.D., Heidenreich O., Fulton D.A. (2013). Determination of Key Structure–Activity Relationships in SiRNA Delivery with a Mixed Micelle System. J. Control. Release.

[46] Chen T., He B., Tao J., He Y., Deng H., Wang X., Zheng Y. (2019). Application of Förster Resonance Energy Transfer (FRET) Technique to Elucidate Intracellular and In Vivo Biofate of Nanomedicines. Adv. Drug Deliv. Rev..

[47] Robin M.P., O’Reilly R.K. (2015). Strategies for Preparing Fluorescently Labelled Polymer Nanoparticles. Polym. Int..

[48] Cauzzo J., Nystad M., Holsæter A.M., Basnet P., Škalko-Basnet N. (2020). Following the Fate of Dye-Containing Liposomes In Vitro. Int. J. Mol. Sci..

[49] Xiao L., Xiong X., Sun X., Zhu Y., Yang H., Chen H., Gan L., Xu H., Yang X. (2011). Role of Cellular Uptake in the Reversal of Multidrug Resistance by PEG-b-PLA Polymeric Micelles. Biomaterials.

[50] Gravier J., Sancey L., Hirsjärvi S., Rustique E., Passirani C., Benoît J.-P., Coll J.-L., Texier I. (2014). FRET Imaging Approaches for In Vitro and In Vivo Characterization of Synthetic Lipid Nanoparticles. Mol. Pharm..

[51] Nguyen H.T.P., Allard-Vannier E., Gaillard C., Eddaoudi I., Miloudi L., Soucé M., Chourpa I., Munnier E. (2016). On the Interaction of Alginate-Based Core-Shell Nanocarriers with Keratinocytes in Vitro. Colloids Surf. B Biointerfaces.

[52] Wolf M.P., Liu K., Horn T.F.W., Hunziker P. (2019). FRET in a Polymeric Nanocarrier: IR-780 and IR-780-PDMS. Biomacromolecules.

[53] Nuhn L., Van Herck S., Best A., Deswarte K., Kokkinopoulou M., Lieberwirth I., Koynov K., Lambrecht B.N., De Geest B.G. (2018). FRET Monitoring of Intracellular Ketal Hydrolysis in Synthetic Nanoparticles. Angew. Chem. Int. Ed..

[54] Thapaliya E.R., Fowley C., Callan B., Tang S., Zhang Y., Callan J.F., Raymo F.M. (2015). Energy-Transfer Schemes To Probe Fluorescent Nanocarriers and Their Emissive Cargo. Langmuir.

[55] Xu Y., Kim C.-S., Saylor D.M., Koo D. (2017). Polymer Degradation and Drug Delivery in PLGA-Based Drug-Polymer Applications: A Review of Experiments and Theories. J. Biomed. Mater. Res. B Appl. Biomater..

[56] Chen H., Kim S., Li L., Wang S., Park K., Cheng J.-X. (2008). Release of Hydrophobic Molecules from Polymer Micelles into Cell Membranes Revealed by Forster Resonance Energy Transfer Imaging. Proc. Natl. Acad. Sci. USA.

[57] Taemaitree F., Fortuni B., Koseki Y., Fron E., Rocha S., Hofkens J., Uji-i H., Inose T., Kasai H. (2020). FRET-Based Intracellular Investigation of Nanoprodrugs toward Highly Efficient Anticancer Drug Delivery. Nanoscale.

[58] Chen K.-J., Chiu Y.-L., Chen Y.-M., Ho Y.-C., Sung H.-W. (2011). Intracellularly Monitoring/Imaging the Release of Doxorubicin from PH-Responsive Nanoparticles Using Förster Resonance Energy Transfer. Biomaterials.

[59] Pu H.-L., Chiang W.-L., Maiti B., Liao Z.-X., Ho Y.-C., Shim M.S., Chuang E.-Y., Xia Y., Sung H.-W. (2014). Nanoparticles with Dual Responses to Oxidative Stress and Reduced PH for Drug Release and Anti-Inflammatory Applications. ACS Nano.

[60] Zhang C., Zhang D., Cheng J.-X. (2015). Coherent Raman Scattering Microscopy in Biology and Medicine. Annu. Rev. Biomed. Eng..

[61] Lai J., Shah B.P., Garfunkel E., Lee K.-B. (2013). Versatile Fluorescence Resonance Energy Transfer-Based Mesoporous Silica Nanoparticles for Real-Time Monitoring of Drug Release. ACS Nano.

[62] Wang D., Chen J., Ren L., Li Q., Li D., Yu J. (2017). AIEgen-Functionalised Mesoporous Silica Nanoparticles as a FRET Donor for Monitoring Drug Delivery. Inorg. Chem. Front..

[63] Elson E.L., Webb W.W. (1974). Fluorescecne Correlation Spectroscopy. II. An Experimental Realization. Biopolymers.

[64] Elson E.L. (1974). Fluorescence Correlation Spectroscopy. I. Conceptual Basis and Theory. Biopolymers.

[65] Silvestri A., Di Silvio D., Llarena I., Murray R.A., Marelli M., Lay L., Polito L., Moya S.E. (2017). Influence of Surface Coating on the Intracellular Behaviour of Gold Nanoparticles: A Fluorescence Correlation Spectroscopy Study. Nanoscale.

[66] Bacia K., Majoul I.V., Schwille P. (2002). Probing the Endocytic Pathway in Live Cells Using Dual-Color Fluorescence Cross-Correlation Analysis. Biophys. J..

[67] Wawrezinieck L., Rigneault H., Marguet D., Lenne P.-F. (2005). Fluorescence Correlation Spectroscopy Diffusion Laws to Probe the Submicron Cell Membrane Organization. Biophys. J..

[68] Tang L., Dong C., Ren J. (2010). Highly Sensitive Homogenous Immunoassay of Cancer Biomarker Using Silver Nanoparticles Enhanced Fluorescence Correlation Spectroscopy. Talanta.

[69] Chen J., Irudayaraj J. (2009). Quantitative Investigation of Compartmentalized Dynamics of ErbB2 Targeting Gold Nanorods in Live Cells by Single Molecule Spectroscopy. ACS Nano.

[70] Capoulade J., Wachsmuth M., Hufnagel L., Knop M. (2011). Quantitative Fluorescence Imaging of Protein Diffusion and Interaction in Living Cells. Nat. Biotechnol..

[71] Shang L., Nienhaus G.U. (2017). In Situ Characterization of Protein Adsorption onto Nanoparticles by Fluorescence Correlation Spectroscopy. Acc. Chem. Res..

[72] Del Pino P., Pelaz B., Zhang Q., Maffre P., Nienhaus G.U., Parak W.J. (2014). Protein Corona Formation around Nanoparticles—From the Past to the Future. Mater. Horiz..

[73] Röcker C., Pötzl M., Zhang F., Parak W.J., Nienhaus G.U. (2009). A Quantitative Fluorescence Study of Protein Monolayer Formation on Colloidal Nanoparticles. Nat. Nanotechnol..

[74] Eriksen A.Z., Brewer J., Andresen T.L., Urquhart A.J. (2017). The Diffusion Dynamics of PEGylated Liposomes in the Intact Vitreous of the Ex Vivo Porcine Eye: A Fluorescence Correlation Spectroscopy and Biodistribution Study. Int. J. Pharm..

[75] Negwer I., Best A., Schinnerer M., Schäfer O., Capeloa L., Wagner M., Schmidt M., Mailänder V., Helm M., Barz M. (2018). Monitoring Drug Nanocarriers in Human Blood by Near-Infrared Fluorescence Correlation Spectroscopy. Nat. Commun..

[76] Schwille P., Meyer-Almes F.J., Rigler R. (1997). Dual-Color Fluorescence Cross-Correlation Spectroscopy for Multicomponent Diffusional Analysis in Solution. Biophys. J..

[77] Meseth U., Wohland T., Rigler R., Vogel H. (1999). Resolution of Fluorescence Correlation Measurements. Biophys. J..

[78] Weidemann T., Schwille P., Roberts G.C.K. (2013). Fluorescence Cross-Correlation Spectroscopy. Encyclopedia of Biophysics.

[79] Mérola F., Nüße O., Dupré-Crochet S., Tramier M., Erard M., Durand D., Bouchab L., Ziegler C.S., Fieschi F. (2019). Quantitative Live-Cell Imaging and 3D Modeling Reveal Critical Functional Features in the Cytosolic Complex of Phagocyte NADPH Oxidase. J. Biol. Chem..

[80] Bacia K., Kim S.A., Schwille P. (2006). Fluorescence Cross-Correlation Spectroscopy in Living Cells. Nat. Methods.

[81] Lakowicz J.R. (2013). Principles of Fluorescence Spectroscopy.

[82] Valeur B. (2001). Molecular Fluorescence: Principles and Applications.

[83] Pacheco-Liñan P.J., Moral M., Nueda M.L., Cruz-Sanchez R., Fernandez-Sainz J., Garzon-Ruiz A., Bravo I., Melguizo M., Laborda J., Albaladejo J. (2017). Study on the PH Dependence of the Photophysical Properties of a Functionalized Perylene Bisimide and Its Potential Applications as a Fluorescence Lifetime Based PH Probe. J. Phys. Chem. C.

[84] Stsiapura V.I., Kurhuzenkau S.A., Kuzmitsky V.A., Bouganov O.V., Tikhomirov S.A. (2016). Solvent Polarity Effect on Nonradiative Decay Rate of Thioflavin, T. J. Phys. Chem. A.

[85] Okabe K., Inada N., Gota C., Harada Y., Funatsu T., Uchiyama S. (2012). Intracellular Temperature Mapping with a Fluorescent Polymeric Thermometer and Fluorescence Lifetime Imaging Microscopy. Nat. Commun..

[86] Romero G., Qiu Y., Murray R.A., Moya S.E. (2013). Study of Intracellular Delivery of Doxorubicin from Poly(Lactide-Co-Glycolide) Nanoparticles by Means of Fluorescence Lifetime Imaging and Confocal Raman Microscopy. Macromol. Biosci..

[87] Dai X., Yue Z., Eccleston M.E., Swartling J., Slater N.K.H., Kaminski C.F. (2008). Fluorescence Intensity and Lifetime Imaging of Free and Micellar-Encapsulated Doxorubicin in Living Cells. Nanomed. Nanotechnol. Biol. Med..

[88] Suarasan S., Craciun A.M., Licarete E., Focsan M., Magyari K., Astilean S. (2019). Intracellular Dynamic Disentangling of Doxorubicin Release from Luminescent Nanogold Carriers by Fluorescence Lifetime Imaging Microscopy (FLIM) under Two-Photon Excitation. ACS Appl. Mater. Interfaces.

[89] Zhou T., Luo T., Song J., Qu J. (2018). Phasor-Fluorescence Lifetime Imaging Microscopy Analysis to Monitor Intercellular Drug Release from a PH-Sensitive Polymeric Nanocarrier. Anal. Chem..

[90] Tasca E., Andreozzi P., Del Giudice A., Galantini L., Schillén K., Maria Giuliani A., de los Ramirez M.A., Moya S.E., Giustini M. (2020). Poloxamer/Sodium Cholate Co-Formulation for Micellar Encapsulation of Doxorubicin with High Efficiency for Intracellular Delivery: An In-Vitro Bioavailability Study. J. Colloid Interface Sci..

[91] Ge H., Cortezon-Tamarit F., Wang H.C., Sedgwick A.C., Arrowsmith R.L., Mirabello V., Botchway S.W., James T.D., Pascu S.I. (2019). Multiphoton Fluorescence Lifetime Imaging Microscopy (FLIM) and Super-Resolution Fluorescence Imaging with a Supramolecular Biopolymer for the Controlled Tagging of Polysaccharides. Nanoscale.

[92] Jeong S., Greenfield D.A., Hermsmeier M., Yamamoto A., Chen X., Chan K.F., Evans C.L. (2020). Time-Resolved Fluorescence Microscopy with Phasor Analysis for Visualizing Multicomponent Topical Drug Distribution within Human Skin. Sci. Rep..

[93] Alex A., Frey S., Angelene H., Neitzel C.D., Li J., Bower A.J., Spillman D.R., Marjanovic M., Chaney E.J., Medler J.L. (2018). In Situ Biodistribution and Residency of a Topical Anti-Inflammatory Using Fluorescence Lifetime Imaging Microscopy. Br. J. Dermatol..

[94] Vanden-Hehir S., Tipping W.J., Lee M., Brunton V.G., Williams A., Hulme A.N. (2019). Raman Imaging of Nanocarriers for Drug Delivery. Nanomaterials.

[95] Li X., Lachmanski L., Safi S., Sene S., Serre C., Grenèche J.M., Zhang J., Gref R. (2017). New Insights into the Degradation Mechanism of Metal-Organic Frameworks Drug Carriers. Sci. Rep..

[96] Klein K., Gigler A.M., Aschenbrenner T., Monetti R., Bunk W., Jamitzky F., Morfill G., Stark R.W., Schlegel J. (2012). Label-Free Live-Cell Imaging with Confocal Raman Microscopy. Biophys. J..

[97] Van Apeldoorn A.A., van Manen H.J., Bezemer J.M., de Bruijn J.D., van Blitterswijk C.A., Otto C. (2004). Raman Imaging of PLGA Microsphere Degradation inside Macrophages. J. Am. Chem. Soc..

[98] Chernenko T., Matthäus C., Milane L., Quintero L., Amiji M., Diem M. (2009). Label-Free Raman Spectral Imaging of Intracellular Delivery and Degradation of Polymeric Nanoparticle Systems. ACS Nano.

[99] Tolstik E., Osminkina L.A., Matthäus C., Burkhardt M., Tsurikov K.E., Natashina U.A., Timoshenko V.Y., Heintzmann R., Popp J., Sivakov V. (2016). Studies of Silicon Nanoparticles Uptake and Biodegradation in Cancer Cells by Raman Spectroscopy. Nanomed. Nanotechnol. Biol. Med..

[100] Iturrioz-rodríguez N., Martín-rodríguez R., Renero-lecuna C., Aguado F., González-legarreta L., González J., Fanarraga M.L. (2021). Applied Surface Science Free-Labeled Nanoclay Intracellular Uptake Tracking by Confocal Raman Imaging. Appl. Surf. Sci..

[101] Lee S., Kwon J.A., Hee K., Min C., Bong J., Choi I. (2018). European Journal of Pharmaceutics and Biopharmaceutics Controlled Drug Release with Surface-Capped Mesoporous Silica Nanoparticles and Its Label-Free in Situ Raman Monitoring. Eur. J. Pharm. Biopharm..

[102] Cialla-May D., Zheng X.S., Weber K., Popp J. (2017). Recent Progress in Surface-Enhanced Raman Spectroscopy for Biological and Biomedical Applications: From Cells to Clinics. Chem. Soc. Rev..

[103] Smith M., Hepel M. (2017). Controlled Release of Targeted Anti-Leukemia Drugs Azacitidine and Decitabine Monitored Using Surface-Enhanced Raman Scattering (SERS) Spectroscopy. Mediterr. J. Chem..

[104] Santiago T., DeVaux R.S., Kurzatkowska K., Espinal R., Herschkowitz J.I., Hepel M. (2017). Surface-Enhanced Raman Scattering Investigation of Targeted Delivery and Controlled Release of Gemcitabine. Int. J. Nanomed..

[105] Kurzątkowska K., Santiago T., Hepel M. (2017). Plasmonic Nanocarrier Grid-Enhanced Raman Sensor for Studies of Anticancer Drug Delivery. Biosens. Bioelectron..

[106] Del Rosal B., Benayas A. (2018). Strategies to Overcome Autofluorescence in Nanoprobe-Driven In Vivo Fluorescence Imaging. Small Methods.

[107] Hong G., Zou Y., Antaris A.L., Diao S., Wu D., Cheng K., Zhang X., Chen C., Liu B., He Y. (2014). Ultrafast Fluorescence Imaging in Vivo with Conjugated Polymer Fluorophores in the Second Near-Infrared Window. Nat. Commun..

[108] Meng F., Wang J., Ping Q., Yeo Y. (2018). Quantitative Assessment of Nanoparticle Biodistribution by Fluorescence Imaging, Revisited. ACS Nano.

[109] Motamarry A., Negussie A.H., Rossmann C., Small J., Wolfe A.M., Wood B.J., Haemmerich D. (2019). Real-Time Fluorescence Imaging for Visualization and Drug Uptake Prediction during Drug Delivery by Thermosensitive Liposomes. Int. J. Hyperth..

[110] Hu Z., Fang C., Li B., Zhang Z., Cao C., Cai M., Su S., Sun X., Shi X., Li C. (2020). First-in-Human Liver-Tumour Surgery Guided by Multispectral Fluorescence Imaging in the Visible and near-Infrared-I/II Windows. Nat. Biomed. Eng..

[111] Zhang H., Fan Y., Pei P., Sun C., Lu L., Zhang F. (2019). Tm 3+-Sensitized NIR-II Fluorescent Nanocrystals for In Vivo Information Storage and Decoding. Angew. Chem..

[112] Li T., Yang D., Zhai L., Wang S., Zhao B., Fu N., Wang L., Tao Y., Huang W. (2017). Thermally Activated Delayed Fluorescence Organic Dots (TADF Odots) for Time-Resolved and Confocal Fluorescence Imaging in Living Cells and In Vivo. Adv. Sci..

[113] Zhang Y., Wei C., Lv F., Liu T. (2018). Real-Time Imaging Tracking of a Dual-Fluorescent Drug Delivery System Based on Doxorubicin-Loaded Globin- Polyethylenimine Nanoparticles for Visible Tumor Therapy. Colloids Surf. B Biointerfaces.

[114] Sun X., Wang G., Zhang H., Hu S., Liu X., Tang J., Shen Y. (2018). The Blood Clearance Kinetics and Pathway of Polymeric Micelles in Cancer Drug Delivery. ACS Nano.

[115] Lu Z., Zhang Z., Tang Y. (2019). Conjugated Polymers-Based Thermal-Responsive Nanoparticles for Controlled Drug Delivery, Tracking, and Synergistic Photodynamic Therapy/Chemotherapy. ACS Appl. Bio Mater..

[116] Zhen S., Yi X., Zhao Z., Lou X., Xia F., Tang B.Z. (2019). Drug Delivery Micelles with Efficient Near-Infrared Photosensitizer for Combined Image-Guided Photodynamic Therapy and Chemotherapy of Drug-Resistant Cancer. Biomaterials.

[117] Du C., Liang Y., Ma Q., Sun Q., Qi J., Cao J., Han S., Liang M., Song B., Sun Y. (2019). Intracellular Tracking of Drug Release from PH-Sensitive Polymeric Nanoparticles via FRET for Synergistic Chemo-Photodynamic Therapy. J. Nanobiotechnol..

[118] Polyak A., Ross T.L. (2018). Nanoparticles for SPECT and PET Imaging: Towards Personalized Medicine and Theranostics. Curr. Med. Chem..

[119] Lee H., Shields A.F., Siegel B.A., Miller K.D., Krop I., Ma C.X., LoRusso P.M., Munster P.N., Campbell K., Gaddy D.F. (2017). 64 Cu-MM-302 Positron Emission Tomography Quantifies Variability of Enhanced Permeability and Retention of Nanoparticles in Relation to Treatment Response in Patients with Metastatic Breast Cancer. Clin. Cancer Res..

[120] Man F., Lammers T., de Rosales T.M.R. (2018). Imaging Nanomedicine-Based Drug Delivery: A Review of Clinical Studies. Mol. Imaging Biol..

[121] Stockhofe K., Postema J., Schieferstein H., Ross T. (2014). Radiolabeling of Nanoparticles and Polymers for PET Imaging. Pharmaceuticals.

[122] Lamb J., Holland J.P. (2018). Advanced Methods for Radiolabeling Multimodality Nanomedicines for SPECT/MRI and PET/MRI. J. Nucl. Med..

[123] Pérez-Medina C., Teunissen A.J.P., Kluza E., Mulder W.J.M., van der Meel R. (2020). Nuclear Imaging Approaches Facilitating Nanomedicine Translation. Adv. Drug Deliv. Rev..

[124] Pérez-Campaña C., Gómez-Vallejo V., Puigivila M., Martín A., Calvo-Fernández T., Moya S.E., Ziolo R.F., Reese T., Llop J. (2013). Biodistribution of Different Sized Nanoparticles Assessed by Positron Emission Tomography: A General Strategy for Direct Activation of Metal Oxide Particles. ACS Nano.

[125] Llop J., Jiang P., Marradi M., Gómez-Vallejo V., Echeverría M., Yu S., Puigivila M., Baz Z., Szczupak B., Pérez-Campaña C. (2015). Visualisation of Dual Radiolabelled Poly(Lactide-Co-Glycolide) Nanoparticle Degradation in Vivo Using Energy-Discriminant SPECT. J. Mater. Chem. B.

[126] Bindini E., de los Ramirez M.A., Rios X., Cossío U., Simó C., Gomez-Vallejo V., Soler-Illia G., Llop J., Moya S.E. In Vivo Tracking of the Degradation of Mesoporous Silica through 89Zr Radio Labelled Core-Shell Nanoparticles. Small.

[127] Tandel H., Bhatt P., Jain K., Shahiwala A., Misra A., Misra A., Shahiwala A. (2018). In-Vitro and In-Vivo Tools in Emerging Drug Delivery Scenario: Challenges and Updates. In-Vitro and In-Vivo Tools in Drug Delivery Research for Optimum Clinical Outcomes.

